# Shared functional microbiome signatures in Parkinson's disease and constipation predominate irritable bowel syndrome despite taxonomic divergence

**DOI:** 10.1016/j.bbih.2026.101218

**Published:** 2026-03-27

**Authors:** Emily C. Hoedt, Grace L. Burns, Kateleen E. Hedley, Sophie Waller, Tamara Canento Sanchez, Olivia Chisolm, Helen MacCallum, Susan Richardson, Belinda Suthers, Elizabeth Pepper, Simon Keely, Nicholas J. Talley

**Affiliations:** a College of Health, Medicine and Wellbeing, University of Newcastle, Callaghan, NSW, Australia; b Centre for Research Excellence in Transforming Gut Health, University of Newcastle, Callaghan, NSW, Australia; c Immune Health Research Program, Hunter Medical Research Institute, New Lambton Heights, NSW, Australia; d Neurology Department, John Hunter Hospital, New Lambton Heights, NSW, Australia; e Department of Neurological Sciences, Prince of Wales Hospital, Randwick, New South Wales, Australia

**Keywords:** Parkinson's disease, Gastrointestinal microbiome, Immune, Microbiome-gut-brain-axis

## Abstract

**Background:**

Gastrointestinal dysfunction, including constipation, is a common non-motor feature of Parkinson's disease (PD) and often precedes motor symptoms. The gut microbiome interacts with the host through neural, hormonal, and immune pathways, yet whether constipation represents a cause or consequence of PD remains unclear. Therefore, we aimed to interrogate the associations between microbiome and immune alterations in relation to constipation to provide novel insight into microbiome-gut-brain axis mechanisms in PD.

**Methods:**

We analysed peripheral blood mononuclear cells (PBMCs) for circulating gut-homing T cell populations and used shotgun metagenomics to profile the stool microbiome composition and functional capacity in PD patients (n = 18), healthy controls (n = 21), and individuals with constipation-predominant irritable bowel syndrome (IBS-C; n = 8). Associations between immune markers and microbial taxa were assessed, and functional pathway differences were evaluated.

**Results:**

Circulating gut-homing T cell frequencies did not differ significantly between PD and controls, but constipated PD patients showed a trend toward increased circulating gut-homing T cells. Microbiome beta-diversity analyses revealed distinct taxonomic shifts in PD and IBS-C, while functional capacity was largely conserved. Of the differential functional pathways tryptophan biosynthesis, polyamine production, and vitamin B metabolism, processes critical for neurotransmitter synthesis, epithelial integrity, and neuroimmune regulation were reduced in PD compared to IBS-C.

**Conclusion:**

Our findings highlight unique microbial and immune signatures in PD, partially overlapping with IBS-C, and underscore the importance of microbial metabolic pathways in gut-brain axis disorders. Collectively our findings suggest a contribution to dopaminergic dysfunction, neuroinflammation, and impaired gut motility. Future longitudinal studies are needed to clarify causal relationships and inform targeted interventions for PD-related gastrointestinal dysfunction.

## Highlights

• PD microbiome shows specific taxonomic and functional changes, not large-scale differences when compared to controls.
• IBS-C and PD share similar functional microbiome profiles, suggesting metabolic outputs may drive overlapping GI symptoms.
• Constipation in PD may be linked to localized immune activation rather than broad immune shifts.

## Glossary

CLR: Centred log-ratio
CNS: Central nervous system
IBS-C: Constipation-predominant irritable bowel syndrome
DHB: Digestive Health's Biobank
DGBI: Disorder of gut brain interaction
GABA: Gamma butyric acid
GI: Gastrointestinal
HADS: Hospital Anxiety and Depression Scale
IBS-SSS: IBS symptom severity scale
MGB: Microbiome-gut-brain
PD: Parkinson's disease
PBMCs: Peripheral blood mononuclear cells
SCFAs: Short chain fatty acids
SNpc: Substantia nigra pars compacta

## 1 Introduction

Parkinson's disease (PD) is a multisystem neurodegenerative disorder characterised by depletion of dopaminergic neurons in the substantia nigra pars compacta (SNpc), and development of intracytoplasmic alpha-synuclein inclusions known as Lewy bodies(Graves et al., 2023). Characteristic non-motor features of PD include constipation, olfactory dysfunction, fatigue and rapid eye movement sleep behavioural disturbance, some of which can precede the onset of motor symptoms by many years(Chaudhuri and Schapira, 2009). Gastrointestinal (GI) manifestations have also been described, including gastroparesis, small intestinal bacterial overgrowth, dyssynergic defaecation, and faecal incontinence(Su et al., 2017). Notably, constipation is a common and often early non-motor symptom of PD, affecting up to two-thirds of individuals with the condition(Arora et al., 2025; Jost, 2010; Sveinbjornsdottir, 2016). In PD, slowed GI transit has been suggested to be a direct result of reduced enteric dopamine signalling and neurodegeneration affecting enteric neurons(Anderson et al., 2007). Constipation not only impacts quality of life (Kenna et al., 2022) but can also interfere with the absorption of medications like levodopa, complicating symptom management(Xu et al., 2022).

The central nervous system (CNS) is no longer recognised as ‘immune privileged’, with substantial evidence for a bidirectional relationship between the CNS and GI system, with intestinal inflammation potentially playing a key role in the pathogenesis of PD. The vagus nerve facilitates this signalling, not only controlling many homeostatic intestinal functions, but may enable transmission of pathogenic alpha-synuclein from the GI tract to the central nervous system(Houser and Tansey, 2017). Furthermore, alpha-synuclein deposition is identified more frequently and in higher amounts throughout the GI tract of patients with PD compared to healthy controls. These demonstrate a rostro-caudal gradient, with the highest concentrations in the submandibular gland and lowest concentrations in the rectum(Houser and Tansey, 2017). Finally, the GI microbiome may influence the onset and progression of a variety of neurodegenerative diseases, and data exist demonstrating an altered microbiome in patients with PD(Scheperjans, 2016; Scheperjans et al., 2015; Zhu et al., 2022). For example, studies have shown that people with PD have different patterns of GI microbiota compared to healthy individuals(Qian et al., 2020). PD GI dysbiosis is characterized by increased putative pro-inflammatory microbes, belonging to the phylum Proteobacteria, and a reduction in putative beneficial short chain fatty acids (SCFAs)-producing bacteria (e.g., *Blautia*, *Roseburia*, and *Faecalibacterium*)(Unger et al., 2016). SCFAs have been shown to have anti-inflammatory and neuroprotective effects and have been linked to improved cognitive function(Qian et al., 2022; Silva et al., 2020). The promotion of a pro-inflammatory GI microbiota profile has also been reported (Marzouk et al., 2025) and as a result an adverse response by the immune system has been documented, affecting the production of key neurotransmitters (neuroinflammation)(Park et al., 2025).

Altered gut microbiota have been reported in chronic gastrointestinal motility disorders such as the irritable bowel syndrome (IBS), a disorder of gut brain interaction (DGBI)(Raskov et al., 2016). With this disorder, for which chronic constipation is a common presentation, patients have been shown to have immune activation and increases in circulating gut-homing T cells that express specific homing receptors, integrins α4 and β7(Burns et al., 2019; Ohman et al., 2009). These features, position IBS patients with predominant constipation symptoms (IBS-C) as a clinically relevant comparator for PD patients experiencing constipation and GI dysfunction.

Although PD and IBS-C are distinct entities, they share overlapping gastrointestinal phenotypes, such as constipation, altered motility, microbiome alterations and evidence of low-grade immune activation. However, their mechanistic underpinnings differ. In PD, enteric dysfunction has been linked to neurodegeneration and reduced dopaminergic signalling, with possible vagal transmission of alpha-synuclein pathology(Anderson et al., 2007; Houser and Tansey, 2017). In IBS-C, cardinal features include dysregulated gut-brain signalling, visceral hypersensitivity and mucosal activation, including expansion of circulating gut-homing lymphocytes(Burns et al., 2019; Liebregts et al., 2007; Ohman et al., 2009). This combination of shared phenotypes and divergent mechanisms provides a powerful framework to determine whether immune signatures and microbial functional pathways are common across constipation-associated conditions or specific to neurodegenerative disease.

Despite growing evidence connecting the microbiome, immune system and gut-brain axis in PD, it remains unclear whether immune signatures characteristic of IBS-C are present in early PD, and how such signatures relate to the gut microbiome. Moreover, comparative analyses that examine functional microbial pathways in addition to taxonomic composition across PD and IBS-C are limited. Addressing this gap may clarify whether overlapping immune-microbiome mechanisms contribute to GI symptoms across disorders or reflect disease-specific processes. As such, the primary question of this study is to identify whether circulating gut-homing T cells are present in the serum of patients with early PD, and to compare the concentration of these lymphocytes in PD patients to healthy controls with the stool microbiome. We aimed to further clarify the link between GI constipation symptoms experienced by PD patients and how the microbiome compares to a group of diagnosed constipation-predominant irritable bowel syndrome individuals.

## 2 Methods

### 2.1 Participant recruitment

PD patients with Hoehn and Yahr stage 1 to 2.5 disease(Hoehn and Yahr, 1967), as diagnosed by a neurologist with expertise in movement disorders, were recruited from the John Hunter Hospital Movement Disorders Clinic. Patients were included in the study if they were aged less than 70 years and scored more than 25 on the Montreal Cognitive Assessment(Julayanont et al., 2015). Control and IBS-C participants had no known neurodegenerative conditions, were aged less than 70 years and were recruited from the Centre for Research Excellence in Digestive Health's Biobank (DHB). IBS-C patients met Rome IV criteria administered as a subsection of the Digestive health and Wellbeing Survey (DHWS5) (Mearin et al., 2016). Exclusion criteria for both patients and controls included antibiotic use in the previous 3 months, a diagnosis of organic gastrointestinal disease (including but not limited to inflammatory bowel diseases and coeliac disease), the presence of a neurodegenerative disorder other than PD, and pregnant or breastfeeding women. All work was carried out with approval from the Hunter New England Local Health District Ethics Committee (references 2018/ETH00317, 2020/ETH01635, 2020/ETH03303).

A medical interview captured demographic data, and non-motor symptoms were assessed in the PD cohort using the PD Non-Motor Symptoms Questionnaire(Chaudhuri et al., 2006). Both patients and controls completed the International Physical Activity Questionnaire (Short Form)(Craig et al., 2003), as well as a validated gastrointestinal outpatient questionnaire. This questionnaire incorporates the modified Talley Bowel Disease Questionnaire(Talley et al., 1989), Rome IV questions for diagnosis of irritable bowel syndrome and functional dyspepsia(Mearin et al., 2016), IBS symptom severity scale (IBS-SSS) (Francis et al., 1997)to assess GI symptoms, as well as the Hospital Anxiety and Depression Scale (HADS) (Zigmond and Snaith, 1983)and questions regarding sleep disturbance.

### 2.2 Sample collection

Samples were recruited from 18 PD patients, 8 IBS-C patients and 21 control subjects. PD and control participants had 30 mL of blood collected in lithium heparin vacutainer tubes for the isolation of plasma and peripheral blood mononuclear cells (PBMCs). PBMCs were isolated as previously described(Burns et al., 2023; Ulmer et al., 1984). Briefly, density gradient centrifugation using lymphoprep media (Stemcell Technologies) was used to fractionate blood samples into plasma and PBMCs within 2 h of sample collection. Plasma aliquots were stored at −80 °C until analysis, while PBMCs aliquots were cryogenically stored in liquid nitrogen until analysis.

In addition, a sample of stool was collected by each participant at home using a stool drop sheet and the Zymo stool preservative kit (Zymo Research). Samples were temporarily stored by the participants in their home freezer (−20 °C) and returned within one week to study researchers where samples were stored at −80 °C until processing.

### 2.3 Flow cytometric analysis of PBMCs

Flow cytometric staining of the cohort occurred over three independent experiments using preserved cytometry settings across all. To revive cells after storage, PBMCs were partially thawed and added dropwise into complete RPMI-1460 media supplemented with 10% foetal calf serum, 1% HEPES, 1% L-glutamine, 1% sodium pyruvate and 1% penicillin-streptomycin. After washing in 1x PBS, the cells were resuspended in complete RPMI-1640 and rested overnight at 37 °C/5% CO_2_ prior to staining. Cells were resuspended in duplicate at 1.5 x 10^6^ cells/well and incubated with a fixable viability dye conjugated to AF700 (BD biosciences catalogue number 564997, 1:1000) for 15 min, followed by an Fc block antibody (BD biosciences, 1:1000) for 10 min at room temperature. Cells were then stained with the following antibodies in a cocktail at 4 °C for 30mins: CD3 BUV805 (BD biosciences catalogue number 612896, 2μL/test), CD4 FITC (BD biosciences catalogue number 555346, 5μL/test), CD8 BUV496 (BD biosciences catalogue number 612942, 2μL/test), CD45RA BUV395 (BD biosciences catalogue number 740315, 2μL/test), CD45RO PE-Cy7 (BD biosciences catalogue number 560608, 2μL/test), integrin ⍺4 PE-CF594 (BD biosciences catalogue number 563645, 5μL/test), integrin β7 BV650 (BD biosciences catalogue number 564285, 5μL/test). Finally, cells were fixed in 4% paraformaldehyde, washed and resuspended in FACS buffer (PBS with 1% FCS and 0.5% EDTA) for overnight storage at 4 °C. Duplicate samples were combined, and surface marker expression was acquired on an LSRFortessa X20 flow cytometer using FACS Diva software (BD Biosciences) set to record 1 x 10^6^ total events. Data was analysed using FlowJo v.10 (BD Biosciences), with live lymphocyte (CD3^+^) populations identified following doublet exclusion **(**Supplementary Table S1**)**.

### 2.4 Microbiome analysis

Total DNA was extracted from patient stool samples using repeated bead beating measures paired with the Promega Maxwell automated DNA recovery system, as previously described(Cuskelly et al., 2022). Metagenomic shotgun sequencing was completed by Microba Pty Ltd (Brisbane, Australia) using the NovaSeq6000 platform, targeting a depth of 4 Gb per sample. Resulting sequence data was processed and analysed as previously described(Hoedt et al., 2024). Briefly this included pre-processing with the KneadData pipeline, taxonomic assignment with MetaPhlAn4 (v4.0.6, database v mpa_vJun23_CHOCOPhlAnSGB_202403) and functional annotation with HUMAnN3 (v3.8), each using default settings.

MetaPhlAn4 taxonomic processed data was visualized in RStudio (R v4.3.3) reporting alpha, beta diversity, and differentially abundant taxa using packages phyloseq (v1.44.0), ampvis2 (v2.8.3), vegan (v2.6-4), SIAMCAT (v2.4.0), microeco (v1.6.0), and microbiome (v1.22.0). Alpha diversity (Chao1, Shannon and Simpson) was analysed for significant differences through Wilcoxon test; Bonferroni correction was used for post-hoc comparisons. Adonis2 (PERMANOVA) was used to test statistical significance of Bray-Curtis PCoA. Associative Spearman analyses with immune and demographic data were then completed with the microeco package. To complete this the data was first normalized with total sum scaling (TSS) and subsequently transformed using centre log ratio (CLR) for rank sum testing.

MetaCyc pathway abundance profiles from HUMAnN3 were used to assess overall functional community structure. Data were normalized and transformed as described above for taxonomic data using R and visualized using packages phyloseq, SIAMCAT, microbiome, and microbiomeutilities (v1.00.16). Pairwise Aitchison (Euclidean CLR) distances were calculated, and PERMANOVA used to determine significant differences. Homogeneity of group dispersions was evaluated using betadisper (vegan). Differentially abundant HUMAnN3 MetaCyc pathways between PD and IBS-C were next identified by Wilcoxon rank sum testing in R using CLR (centred log-ratio) transformed data.

### 2.5 Statistical analysis

Demographic and immunological data were analysed using Graphpad Prism 10 (Graphpad Software Inc, La Jolla, USA) and visually presented as mean ± SEM, with statistics reported as mean ± SD. Distribution normality was assessed for all datasets using the D'Agostino-Pearson test. Group demographics were compared with Fisher's exact test. Comparisons of the peripheral immune data between groups were made using t tests or one-way ANOVA with uncorrected Fisher's LSD post-hoc test for normally distributed data, while Mann-Whitney t tests or Kruskal-Wallis test with Dunn's multiple comparisons post-hoc tests were used for non-normally distributed data. Outliers were identified within immune data using Grubb's outlier test, however, were not removed from analyses due to low sample numbers and expected biological variation between subjects, except where specified within text. Statistical significance was considered *p* < 0.05.

### 2.6 Data availability

Shotgun metagenomic raw sequence data are available and have been deposited under NCBI BioProject accession number PRJNA1363568. NCBI SRA record is accessible with the following link http://www.ncbi.nlm.nih.gov/bioproject/PRJNA1363568.

## 3 Results

### 3.1 Cohort characteristics

In total, 18 PD patients and 21 control subjects were recruited to this study, with stool microbiome available for 20 controls (59 ± 10.97 years, 11 female) and 18 PD patients (59.78 ± 5.80 years, 12 female; Table 1). PBMC samples were available for 21 controls (58.96 ± 12.36 years, 11 female) and 13 PD patients (61.46 ± 5.38 years, 7 female; Table S2).

**Table 1:** Cohort characteristics of controls and PD patients with microbiome samples available. Group demographics were compared with Fisher's exact test.

	Control (n = 20)	Parkinson's Disease (n = 18)	P-value (PD vs Control)	IBS-C (n = 8)	P-value (PD vs IBS-C)
Age (mean ± SD)	59 ± 10.97	59.78 ± 5.80	0.98	59.63 ± 8.80	>0.99
Sex (female, %)	11 (55%)	12 (61%)	0.52	7 (87.5%)	0.38
BMI (mean ± SD)	26.30 ± 4.78	25.76 ± 5.36	0.71	27.26 ± 4.89	0.47
Difficulty swallowing (%)	0	7 (39%)	**0.003**		
Vomiting or nausea (%)	0	7 (39%)	**0.003**		
Constipation in last month (%)	3 (15%)	7 (39%)	0.14	8 (100%)	**0.007**
Incomplete bowel emptying (%)	0	8 (44%)	**0.0009**		

**Bowel Pattern in the last 12 months**^a^					

Normal (%)	20 (100%)	9 (53%)	**0.0006**		
Alternating (%)	0	2 (12%)	0.2		
Constipated (%)	0	6 (35%)	**0.005**	8 (100%)	**0.003**

**Smoking history**					

Non-smoker (%)	15 (75%)	11 (61%)	0.49	6 (75%)	0.67
Ex-smoker (%)	4 (0.2)	7 (39%)	0.29	2 (25%)	0.67
Current smoker (%)	1 (0.05%)	0	>0.99	0	>0.99

a Data not reported for all; body mass index (BMI); standard deviation (SD); Constipation defined as answering yes to the question “less than 3 bowel movements a week or having to strain to pass a stool”.

There were no differences in age, sex or BMI between controls and patients. Constipation was assessed with two different questionaries over the past year (outpatient questionnaire “bowel pattern”) and past month (PD NMS questionnaire “constipation”; Table 1), defined as <3 bowel movements a week-or having to strain to pass a stool. Of the participants with microbiome samples available, over the past year none of the controls reported constipation while in the past month leading up to the provision of microbiome sample 15% of controls reported constipation. In contrast 47% of PD patients reported alternating/constipation bowel pattern in the past year and 39% reported constipation in the month leading up to sample provision (Table 1). The constipation bowel habits for the last year was significant when comparing controls to PD (35%; *p* = 0.005). A further eight IBS-C were included for microbiome comparison to PD patients (Table 1). There were no differences in age, sex or BMI between PD and IBS-C. Clinical characteristics and medications for PD participants at the time of sample collection are detailed in Table S3.

### 3.2 CD4^+^ and CD8^+^ T cell sub-populations are unchanged in PD patients compared to controls

**Fig. 1:**
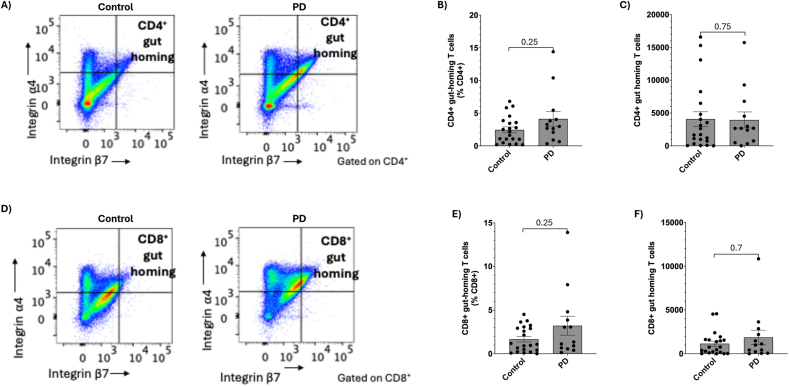
Circulating gut-homing T cells in peripheral blood from controls and Parkinson's Disease (PD) patients. **A)** Peripheral blood mononuclear cells were isolated from whole blood and the proportions of integrin ⍺4^+^ integrin β7^+^ cells were assessed within the CD4^+^ population as both **B)** a proportion and **C)** total cell number. **D)** Gut-homing cells were also assessed within the total CD8^+^ population as both **E)** a proportion of CD8^+^ cells and **F)** total cell number. n = 21 controls, n = 13 PD, data presented as mean ± SEM.

We first sought to characterise the peripheral lymphocyte populations in PBMCs isolated from PD patients compared to controls via flow cytometry (Fig. S1A). We found no significant differences between the proportion (*p* = 0.11; Fig. S1B) or total cell number (*p* = 0.12; Fig. S1C) of viable CD3^+^ lymphocytes. There was also no difference between T helper cell proportion (*p* = 0.61; Fig. S1D) or total number (*p* = 0.09; Fig. S1E), nor in proportion (*p* = 0.77; Fig. S1F) or total number (*p* = 0.3; Fig. S1G) of cytotoxic T cells between PD patients and controls. We next examined the expression of integrins ⍺4 and β7 as indicators of lymphocytes with a circulating gut-homing phenotype within the CD4^+^ population (Fig. 1A). There was no significant difference in the proportion (*p* = 0.25; Fig. 1B) or total number (*p* = 0.75; Fig. 1C) of CD4^+^ circulating gut-homing T cells. We performed the same analysis on the CD8^+^ population, again finding no change between the proportion (*p* = 0.25; Fig. 1E) or total number (*p* = 0.7; Fig. 1F) in PD patients compared to controls.

We next sought to examine the populations of naïve and effector CD4^+^ lymphocytes in PD patients compared to controls (Fig. S2A), finding no differences in the proportions (*p* = 0.65, *p* = 0.49 respectively; Fig. S2B and S2D) or total cell numbers (*p* = 0.14, *p* = 0.97 respectively; Fig. S2C and S2E) of either population. Similarly, we saw no changes in the CD4^+^ central or effector memory populations (Fig. S2F) by proportion of total CD4^+^ T cells (*p* = 0.68, *p* = 0.67 respectively; Fig. S2G and S2I) or total cell number (*p* = 0.1, *p* = 0.86 respectively; Fig. S2H and S2J)

We also investigated the naïve and effector populations within the CD8^+^ lymphocyte pool (Fig. S3A), again finding no differences in the proportions (*p* = 0.42, *p* = 0.24 respectively; Fig. S3B and S3D) or total cell numbers (*p* = 0.33, *p* = 0.6 respectively; Fig. S3C and S3E) of either population. Similarly, we saw no changes in the CD8^+^ central or effector memory populations (Fig. S3F) by proportion of total CD8^+^ T cells (*p* = 0.8, *p* = 0.47 respectively; Fig. S3G and S3I) or total cell number (*p* = 0.42, *p* = 0.24 respectively; Fig. S3H and S3J). Collectively, these findings suggest the overall circulating lymphocyte pool is unchanged between PD patients and controls.

### 3.3 There may be a relationship between CD8^+^ circulating gut-homing T cells in PD patients who report constipation compared to those who do not

**Fig. 2:**
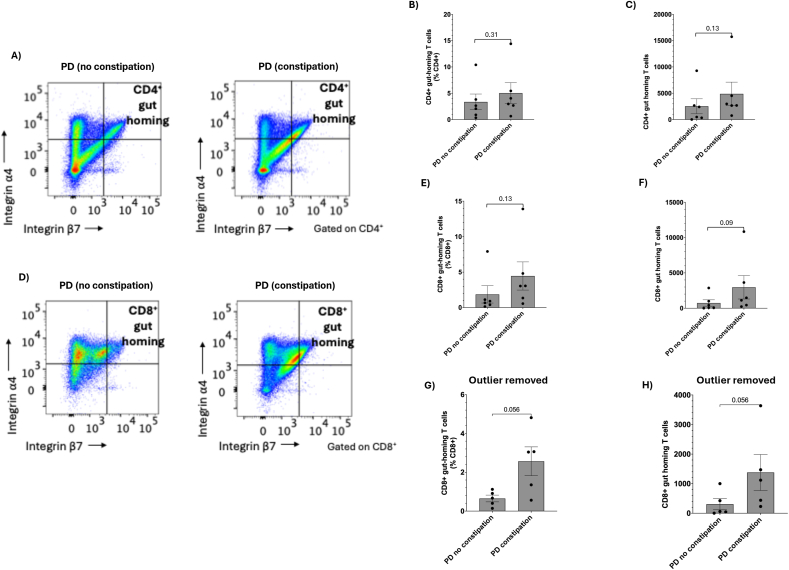
CD4^+^ and CD8^+^ circulating gut homing T cells in Parkinson's Disease (PD) patients with constipation compared to those without. **A)** Peripheral blood mononuclear cells were isolated from whole blood and the proportions of integrin ⍺4^+^ integrin β7^+^ cells were assessed within the CD4^+^ population as both **B)** a proportion and **C)** total cell number. **D)** Gut-homing cells were also assessed within the total CD8^+^ population as both **E)** a proportion of CD8^+^ cells and **F)** total cell number for all samples. Identification of n = 1 outlier per group by Grubbs outlier test results for CD8^+^ circulating gut homing cells as **(G)** a proportion of CD8^+^ cells and **(H)** total cell number. n = 6 no constipation, n = 6 with constipation, data presented as mean ± SEM.

The PD patient cohort was then split into those reporting constipation (defined as less than 3 bowel movements a week or having to strain to pass a stool, n = 6) and those without constipation (n = 6) to examine CD4^+^ circulating gut-homing T cells (Fig. 2A). There was no difference in the proportion (*p* = 0.31; Fig. 2B) or total cell number (*p* = 0.13; Fig. 2C) within the CD4^+^ pool. We also examined circulating gut-homing within the CD8^+^ population (Fig. 2D), initially finding no difference between the proportion (1.86 ± 2.99 vs 4.45 ± 4.86, *p* = 0.13; Fig. 2E) or total cell number (732.7 ± 1110 vs 2956 ± 4051, *p* = 0.09; Fig. 2F). However, within each group, exclusion of statistically significant outliers (n = 1 per group) identified a trend towards an increase in both the proportion (0.65 ± 0.39 vs 2.56 ± 1.66, *p* = 0.056) (Fig. 2G) and total number (306.6 ± 423.8 vs 1378 ± 1358, *p* = 0.056; Fig. 2H) of CD8^+^ circulating gut-homing T cells in those with constipation compared to those without. This data would suggest the study is underpowered for this sub-analysis.

### 3.4 Gut microbiome and immune correlative profile is distinct from that of healthy controls

**Fig. 3:**
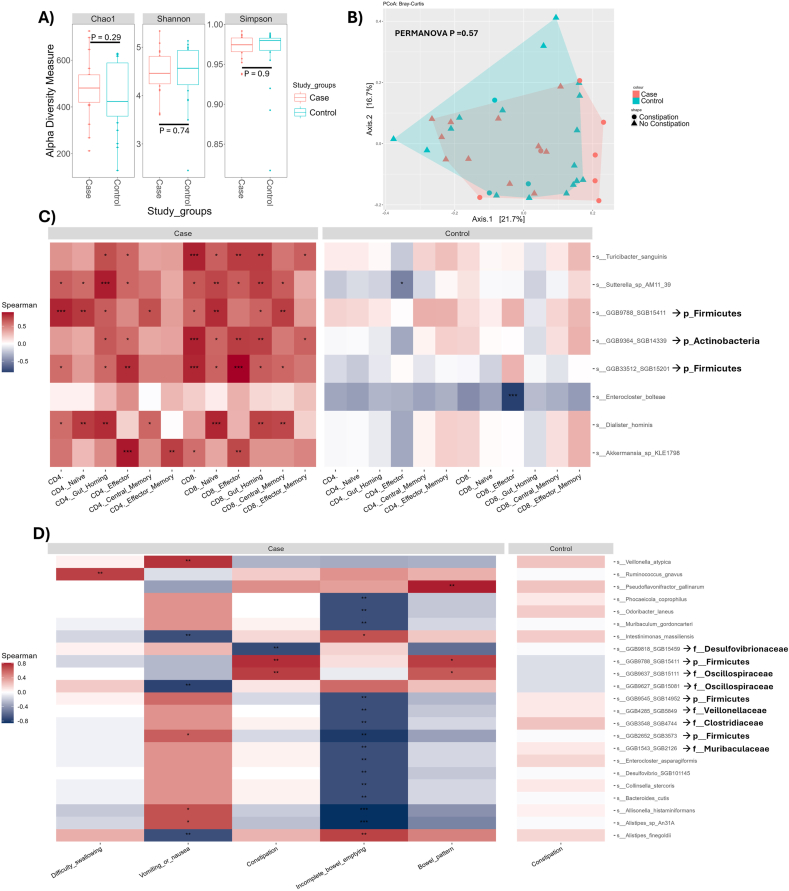
**A)** Alpha diversity indices Chao1, Shannon and Simpson of Parkinson's Disease cases (red) and healthy controls (blue). **B)** Beta diversity PCoA Bray-Curtis plot comparing Parkinson's cases (red) and healthy controls (blue). Spearman correlations for stool microbiome species with **C)** total PBMC cell counts of effector and memory T cell types quantified with flow cytometry, and **D)** self-report gastrointestinal symptoms. Red denotes a positive correlation and blue a negative correlation. Adjusted P-values <0.05 were considered significant; ∗P < 0.05, ∗∗P < 0.01, ∗∗∗P < 0.001. Alpha diversity was analysed for significant difference through Wilcoxon test and beta diversity significance assessed by PERMANOVA P-values <0.05 were considered significant. Error bars represent standard deviation. Data for spearman correlation was transformed with centre log ratio (CLR) and species correlations with at least one ∗ and one ∗∗ are represented. (For interpretation of the references to colour in this figure legend, the reader is referred to the Web version of this article.)

We next compared the gut microbiome composition of PD patients and healthy controls. No significant difference was observed when comparing the alpha diversity indices for Chao1, Shannon or Simpson (*p* = 0.29, 0.74, 0.9, respectively; Fig. 3A). Furthermore, when beta diversity was assessed by Bray-Curtis PCoA there was no significant difference in the clustering of the groups (PERMANOVA *p* = 0.57; Fig. 3B). However, when the gut microbiome taxonomic profiles were correlated with the PBMC effector and memory T cell types quantified with flow cytometry a distinct profile was apparent for PD patients in comparison to healthy controls. Notably healthy controls had only two significant negative associations with measured immune markers, this included *Sutterella* sp. AM11_39 with CD4 Effector cells (rho = −0.507, *p* = 0.040) and *Enterocloster bolteae* with CD8 Effector cells (rho = −0.811, *p* < 0.001; Fig. 3C). In contrast PD patients had seven microbial species that were positively associated with several of the assessed PBMC cell types. These included *Turicibacter sanguinis* (rho = 0.841 with CD8^+^ T cells, *p* = 0.001), *Sutterella* sp. AM11_39 (rho = 0.852 with CD4 circulating gut-homing cells, *p* < 0.001), GGB9788 SGB15411 (rho = 0.835 with CD4^+^ T cells, *p* = 0.001), GGB9364 SGB14339 (rho = 0.846 with CD8^+^ T cells, *p* < 0.001), GGB33512 SGB15201 (rho = 0.896 with CD8 Effector cells, *p* < 0.001), *Dialister hominis* (rho = 0.830 with CD8 Naïve cells, *p* = 0.001), and *Akkermansia* sp. KLE1798 (rho = 0.863 with CD4 Effector cells, *p* < 0.001). Full correlation results for all species-immune marker combinations are provided in Supplementary Table S4.

### 3.5 GI symptoms and gut microbiome associations distinguish PD

Constipation is a prevalent and burdensome symptom of PD(Xu et al., 2022), therefore, we investigated the gut microbiome associations with reported gastrointestinal symptoms of this cohort to determine if any of the immune associated species are conserved. Interestingly, when examining the gut microbiome associations with the two different constipation reporting metrics we observed the same associative profile for PD patients (Fig. 3D). For example, several species showed moderate-to-strong associations with constipation, including GGB9818 SGB15459 (rho = −0.72, *p=*0.006), GGB9788 SGB15411 (rho = 0.77, *p* = 0.003), and GGB9637 SGB15111 (rho = 0.72, *p=*0.006). Furthermore, “incomplete bowel emptying” produced a similar but broader profile to that of “bowel pattern”, with several taxa displaying moderate correlations (rho ∼ ±0.71, *p* = 0.006), and stronger associations observed for *Allisonella histaminiformans* (rho = −0.86, *p* < 0.001) and *Alistipes* sp. An31A (rho = −0.86, *p* < 0.001).

Other symptom domains showed distinct associations. “Difficulty swallowing” was positively associated with *Ruminococcus gnavus* (rho = 0.72, *p* = 0.008). “Vomiting or nausea” displayed a mixture of positive and negative correlations, including *Veillonella atypica* (rho = 0.76, *p=*0.004), *Intestinimonas massiliensis* (rho = −0.71, *p* = 0.006), GGB9627 SGB15081 (rho = −0.76, p = 0.004), and GGB2652 SGB3573 (rho = 0.61, *p* = 0.02). In contrast, controls only produced a non-significant profile for constipation reported over the last year and this was different to that seen in PD patients (Fig. 3D). Full correlation results are provided in Supplementary Table S4.

### 3.6 While microbial profiles are distinct between PD and IBS-C, functional analysis suggests significant overlap between diseases

**Fig. 4:**
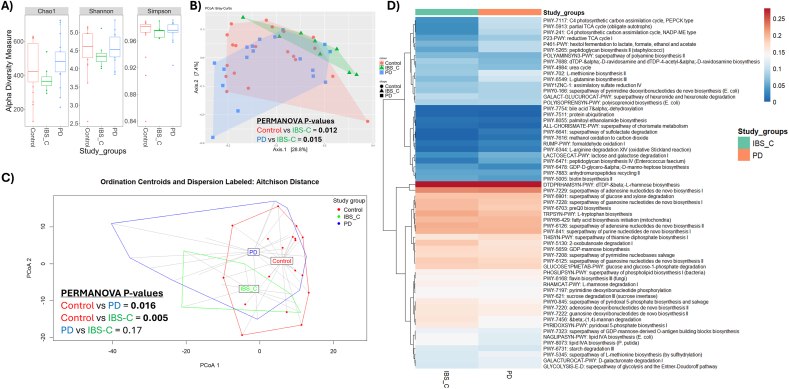
**A)** Taxonomic alpha diversity indices Chao1, Shannon and Simpson of Parkinson's disease cases, irritable bowel syndrome-constipation (IBS-C) and healthy controls. **B)** Taxonomic beta diversity PCoA Bray-Curtis plot comparing Parkinson's disease cases, IBS-C and healthy controls. **C)** Ordination of CLR-transformed MetaCyc functional pathway abundances based on Aitchison (Euclidean CLR) distance to visualize overall differences in functional composition between study groups (Control, IBS-C, and PD). Group centroids and dispersion ellipses (95% confidence) were derived from multivariate homogeneity of dispersion analysis. Colours denote study groups (Control = red, IBS-C = green, PD = blue). **D)** Heatmap of significantly different MetaCyc pathways between Parkinson's disease (PD) and constipation predominant irritable bowel syndrome (IBS-C) groups. MetaCyc pathway abundances derived from HUMAnN3 were CLR-transformed, and differential abundance was assessed using Wilcoxon rank-sum tests. The 60 pathways with significant differences (*adjusted p* < 0.05) are shown, scaled by row Z-score to highlight relative enrichment patterns across groups. Alpha diversity was analysed for significant difference through Wilcoxon test and beta diversity significance assessed by PERMANOVA P-values <0.05 were considered significant. Error bars represent standard deviation. (For interpretation of the references to colour in this figure legend, the reader is referred to the Web version of this article.)

Analysis of taxonomic alpha diversity indices (Chao1, Shannon and Simpson) revealed no significant differences between control, PD, and IBS-C groups (Fig. 4A). Beta diversity analysis indicated significant taxonomic compositional differences between PD and IBS-C (PERMANOVA P = 0.015), and between controls and IBS-C (PERMANOVA *p* = 0.012; Fig. 4B). Next, we investigated the differences in the functional capacity of each group (Fig. 4C). Ordination of CLR-transformed MetaCyc functional pathway abundances showed significant differences in functional composition between PD and controls (PERMANOVA *p* = 0.016), and IBS-C and controls (PERMANOVA *p* = 0.005). Interestingly, we did not observe a significant difference in the functional capacity between PD and IBS-C (PERMANOVA *p* = 0.17). Between PD and IBS-C 60 differentially abundant pathways were significant, of these 14 were significantly increased in PD compared to IBS-C (Fig. 4D). Notably, a number of pathways that are known to influence the brain were significantly decreased in PD compared to IBS-C. These included neurotransmitters L-tryptophan biosynthesis (TRPSYN-PWY), pyridoxal 5 phosphate biosynthesis I (PYRIDOXSYN PWY), superpathway of pyridoxal 5 phosphate biosynthesis and salvage (PWY0 845), pyridoxal is a cofactor in neurotransmitter synthesis (e.g., GABA, dopamine), and palmitoylethanolamide biosynthesis (PWY-8055) which is reported to have neuroprotective properties(Di Stefano et al., 2025).

## 4 Discussion

The onset of non-motor features, such as alterations in GI function, are common in early PD(Abbott et al., 2001; Arora et al., 2025; Jost, 2010; Sveinbjornsdottir, 2016). In fact, several studies have highlighted the co-occurrence of constipation in PD which are postulated to be a direct result of changes in intestinal function and structure or in associated neural structures(Anderson et al., 2007). If we further consider the complicated nature of the GI tract encompassing the microbiome's interaction with the host through neural, hormonal and immune crosstalk the question of constipation cause or consequence remains unanswered. Here we aimed to examine alterations in PD patients' peripheral immune profiles in association with specific changes in the GI tract microbiome. Furthermore, given the high prevalence of constipation in PD patients, we compared the GI microbiome of PD to individuals diagnosed with IBS-C to explore the taxonomic and functional differences between these groups to isolate the role of PD microbiome in modulating the gut-brain axis and their specific non-motor symptoms (i.e., mood, cognition, and bodily functions). While no significant differences in circulating gut-homing T cell populations were observed between PD and controls, a trend toward increased circulating gut-homing T cells was noted in constipated PD patients. This suggests that GI symptoms in PD may be associated with localized immune activation, potentially driven by altered microbial exposure or mucosal barrier disruption. Indeed, we observed a significantly altered microbial associative profile between PD and controls peripheral immune markers, although the alpha and beta diversity were not significantly different between these groups suggesting that there are specific species associated with PD rather than large community changes. Finally, we interrogated the functional capacity of the GI microbiome, and included a group of individuals with diagnosed IBS-C. Interestingly, while the taxonomic composition of the IBS-C group was significantly different to that of PD and controls, the IBS-C functional capacity was not significantly different to that of PD. This conserved functional capability despite taxonomic divergence between IBS-C and PD suggests that the GI microbiome metabolic outputs, rather than specific microbes, may underpin overlapping GI symptoms across these conditions.

Increasing evidence supports the bidirectional communication between the GI microbiome and the CNS, and this microbiome-gut-brain (MGB) axis likely plays a role in cognition and mental health, with disruptions of the microbiome being implicated in several neurological disorders(Morais et al., 2020; Rhee et al., 2009). The mechanisms driving these associations are not well understood but ongoing research has identified multiple pathways that intersect the MGB axis, including bioactive signalling molecules(Dalile et al., 2019; Giri et al., 2022; Needham et al., 2020), neurotransmitters that are produced or metabolized by the gut microbiome(Strandwitz, 2018) and modulation of both enteric nerve signalling and stress as a result of host-microbe interactions(Foster et al., 2017). IBS-C is classified as a disorder of gut brain interaction and the GI symptoms experienced between the two conditions have significant overlap. Further, IBS is reported to be a risk factor for later development of PD(Abbott et al., 2001; Konings et al., 2023; Savica et al., 2009).

Therefore, when we investigated the microbial functional overlap between these groups, it was of interest that, while ordination of CLR-transformed functional pathways showed no significant difference in the overall functional composition between IBS-C and PD, several microbial pathways were significantly decreased in PD, each with direct implications for the MGB axis. Pathways involved in tryptophan biosynthesis, pyridoxal 5-phosphate metabolism and polyamine production were shown the be decreased in the PD cohort. Tryptophan is a key component of the serotonin and melatonin synthesis pathways, which are known to be dysregulated in both conditions and can impact GI motility(Gabela et al., 2026; Höglund et al., 2019). Serotonin increases gut motility and promotes peristaltic reflexes, and a reduction in serotonin has been linked to constipation in IBS-C. This could potentially be a contributing cause to the constipation seen in PD. Additionally in PD, alterations to serotonin levels are thought to contribute to many of the other non-motor symptoms, such as fatigue, sleep disturbances, anxiety and depression(Wilson et al., 2018), whereas in IBS-C they may perpetuate visceral hypersensitivity and impaired barrier function(Atkinson et al., 2006). In addition, tryptophan can be converted into kynurenine, which is then metabolized to neuroactive compounds quinolinic acid and kynurenic acid(Höglund et al., 2019). It has been shown that dysregulation on this pathway is associated with peripheral and neuroinflammation(Wilson et al., 2025). The identification of these microbial pathways clearly highlights the potential link between GI dysregulation and the underlying causes of both motor and non-motor symptoms in PD, and the metabolic, inflammatory and neuropathological links.

Pyridoxal 5-phosphate is the active form of vitamin B6 and is a coenzyme in over 100 enzymatic reactions, playing a crucial role in amino acid metabolism and production of multiple neurotransmitters, such as serotonin and dopamine(Parra et al., 2018). Most relevant to PD, pyridoxal 5-phosphate is a necessary cofactor for the conversion of L-DOPA into dopamine(Parra et al., 2018). The inefficient conversion of L-DOPA to dopamine leads to the unstable dopamine levels and the exacerbation of motor symptoms (i.e., on/off periods). In addition, deficiency of pyridoxal 5-phosphate have been linked to seizures in PD(Pulluru et al., 2024), due to unstable levels of the inhibitory neurotransmitter gamma butyric acid (GABA) in the CNS. Similarly, polyamines and vitamin B6 derivatives support neuronal health and immune regulation, while alterations in these pathways may exacerbate constipation and systemic inflammation. Vitamin B-related pathways are highly relevant in the context of PD because these vitamins play critical roles in both neuronal and GI health. Biotin (vitamin B7) and riboflavin (vitamin B2) are essential cofactors in energy metabolism and mitochondrial function, processes that are impaired in PD due to dopaminergic neurodegeneration(Lai et al., 2025; Tao et al., 2025). Similarly, thiamine (vitamin B1) is required for neurotransmitter synthesis and carbohydrate metabolism, influencing dopaminergic signalling and GI motility(Mrowicka et al., 2023). Alterations in microbial pathways for these vitamins could therefore impact host vitamin availability, contributing to neuroinflammation, oxidative stress, and GI dysfunction observed in PD. In IBS-C, microbial vitamin metabolism may affect mucosal integrity and immune regulation, potentially exacerbating symptoms, including constipation. These findings underscore the importance of considering microbial vitamin biosynthesis as a mechanistic link in MGB disorders.

The immune system and GI microbiome components directly modulate MGB axis function both locally and systemically, contributing to neurological function and behaviour(Dalile et al., 2019; Sylvia and Demas, 2018). PD patients often present with chronic low-grade inflammation, including elevated pro-inflammatory cytokines and activated microglia in the brain, which may contribute to neurodegeneration(Tansey et al., 2022). In the GI tract, immune dysregulation is reflected by increased permeability and altered mucosal immunity(Vanheel et al., 2014), potentially allowing microbial antigens to trigger systemic immune activation. Although not significant, our data showing a trend toward higher circulating gut-homing T cells in constipated PD patients aligns with this concept, suggesting that GI dysfunction may amplify immune signalling. Although there is a lack of adequately powered studies assessing immune parameters in IBS (specifically circulating gut-homing T cells), a systematic review reported the increased proportions of peripheral gut-homing T cells of IBS patients(Burns et al., 2019). This observation would support the hypothesis that disruption of mucosal homeostasis may contribute to gastrointestinal symptoms. Furthermore, these immune changes could link gut dysbiosis to central neuroinflammation via the MGB axis, reinforcing the hypothesis that peripheral immune activation is not merely a consequence but may be an early contributor to PD progression.

Previous studies have reported alterations in circulating T cell phenotypes in PD, notably reduced CD8^+^ T cells and specific auto-reactive T cells against ⍺-synclein and phosphatase and tensin homolog (PTEN)-induced kinase 1 (PINK1) (Johansson et al., 2025). The reduction in the CD8^+^ population has been shown to correlate with disease severity(Bhatia et al., 2021), and this is likely why we did not observe this shift, given our inclusion criteria specified patients must present with early to mid-stage disease. Importantly, given circulating immune cells are not reflective of compartmentalised tissue specific T cell populations within the GI tract, further profiling of these intestinal populations is critical to define relationships between GI symptoms and disease progression in PD.

There are some limitations to this study, namely the sample size of our recruited groups is underpowered, however, this was designed as an exploratory study to highlight areas worthy of further investigation. Furthermore, the role of dopaminergic therapy in modulating GI function further complicates interpretation(Serio and Zizzo, 2023). All PD patients reporting constipation were on some form of levodopa, which is known to influence GI motility through dopamine receptor activation. However, previous studies suggest that plasma levodopa levels, rather than medication intake alone, are more closely associated with GI motility(Jost, 2010). This raises the possibility that both disease-related neurodegeneration and treatment effects contribute to altered GI function and immune responses in PD. Moreover, emerging biomarkers such as DOPA decarboxylase may help disentangle the contributions of dopaminergic cell loss from medication effects in future studies(Verbeek and Bloem, 2023). Another major consideration for the work presented here is that our study focuses on associations between GI microbiome, immune cell populations, and GI symptoms and does not provide direct mechanistic evidence, however, our findings suggest potential interactions worthy of further investigation. These findings underscore the importance of microbial metabolic capacity as a mechanistic link between gut dysbiosis and neurological and/or GI symptoms.

In conclusion, we have provided new evidence to support a complex interplay between gut microbiome composition, microbial functional capacity, and immune activation in PD, with partial constipation overlap. These findings highlight that while constipation is a shared symptom, the underlying mechanisms differ substantially between these conditions, with PD showing unique signatures in immune reactivity and microbial metabolic pathways relevant to neurotransmission and vitamin biosynthesis. Overall, our study emphasizes the need for longitudinal, multi-omic approaches to disentangle the causal relationships between GI microbiome functional imbalances, immune activation, and neurodegeneration, which may ultimately inform targeted interventions for PD.

## CRediT authorship contribution statement

**Emily C. Hoedt:** Writing – review & editing, Writing – original draft, Visualization, Project administration, Methodology, Investigation, Funding acquisition, Formal analysis, Data curation, Conceptualization. **Grace L. Burns:** Writing – review & editing, Writing – original draft, Visualization, Methodology, Investigation, Funding acquisition, Formal analysis, Data curation, Conceptualization. **Kateleen E. Hedley:** Writing – review & editing, Writing – original draft. **Sophie Waller:** Writing – review & editing, Data curation. **Tamara Canento Sanchez:** Writing – review & editing, Data curation. **Olivia Chisolm:** Writing – review & editing, Data curation. **Helen MacCallum:** Writing – review & editing, Data curation. **Susan Richardson:** Writing – review & editing, Data curation. **Belinda Suthers:** Writing – review & editing, Data curation. **Elizabeth Pepper:** Writing – review & editing, Data curation, Conceptualization. **Simon Keely:** Writing – review & editing, Supervision, Resources, Conceptualization. **Nicholas J. Talley:** Writing – review & editing, Supervision, Resources, Conceptualization.

## Declaration of generative AI and AI-assisted technologies in the manuscript preparation process

During the preparation of this work the authors used M365 Copilot to improve the language of some sections. After using this tool/service, the authors reviewed and edited the content as needed and takes full responsibility for the content of the published article.

## Funding sources

The work was funded by a University of Newcastle strategic support grant, a College of Health, Medicine and Wellbeing Strategic Research Pilot Grant Scheme, and the Centre of Research Excellence in Digestive Health from the NHMRC. ECH is supported by the Australian NSW Health Round 5 Early-Mid Career Grant.

## Declaration of competing interest

The authors declare the following financial interests/personal relationships which may be considered as potential competing interests: Emily C. Hoedt reports financial support was provided by The University of Newcastle. Grace L. Burns reports financial support was provided by The University of Newcastle. Simon Keely reports a relationship with University of Newcastle that includes: consulting or advisory. Nicholas J. Talley reports a relationship with The University of Newcastle that includes: consulting or advisory. Nicholas J. Talley has patent licensed to MAPI. Simon Keely (SK) reports consultancy and positions held on advisory boards for: Gossamer Bio (Scientific Advisory Board), Anatara Lifescience (Scientific Advisory Board), Microba Life Science (Consultancy) and Immuron Ltd. (Consultancy).

Nicholas J Talley (NJT) reports personal fees from Brown University & US Agency for Health Care Research and Quality (fiber and laxation), Rome Foundation (member gastroduodenal committee), Biocodex (FD diagnostic tool), Microba (microbiome), Comvita Manuka Honey (FD trial consulting), BluMaiden (microbiome), Schwabe (FD/IBS) outside the submitted work. In addition, Dr. Talley has Licensing Questionnaires: Nepean Dyspepsia Index (NDI) 1998 licensed to MAPI, Talley Bowel Disease Questionnaires licensed to Mayo Clinic. Patents: “Performance of a biomarker panel for irritable bowel syndrome” EP2710383B1 “Diagnostic marker for functional gastrointestinal disorders” US Patent Application No. 20240272175A1 (provisional), “Methods and compositions for treating age-related neurodegenerative disease associated with dysbiosis” US Patent Application No. 63/537,725, “Compositions and methods for the treatment of oesophageal disorders” Australian Provisional Patent ID 2025902002.

If there are other authors, they declare that they have no known competing financial interests or personal relationships that could have appeared to influence the work reported in this paper.

## Data Availability

Shotgun metagenomic raw sequence data are available and have been deposited under NCBI BioProject accession number PRJNA1363568. NCBI SRA record is accessible with the following link http://www.ncbi.nlm.nih.gov/bioproject/PRJNA1363568.
