# Supplementary material for: Shared functional microbiome signatures in Parkinson's disease and constipation predominate irritable bowel syndrome despite taxonomic divergence
Source: Brain Behav Immun Health. 2026 Mar 27;53:101218. doi: 10.1016/j.bbih.2026.101218 (PMC13081680; doi:10.1016/j.bbih.2026.101218)
Supplement: Multimedia component 1 [file mmc1.docx]

**Supplementary Tables and Figures**

**Supplementary Table S1:** Surface markers for the identification of T cell subsets in peripheral blood mononuclear cells.

| **T cell subset** | **Surface marker phenotype** |
| --- | --- |
| Viable lymphocytes | Fixable viability stain 700^-^ CD3^+^ |
| T helper cells | CD3^+^ CD4^+^ |
| Cytotoxic T cells | CD3^+^ CD8^+^ |
| Gut-homing T cells | CD4^+^ integrin ⍺4^+^ integrin β7^+^ / CD8^+^ integrin ⍺4^+^ integrin β7^+^ |
| Naïve T cells | CD4^+^ CD45RA^+^ CCR7^+^ / CD8^+^ CD45RA^+^ CCR7^+^ |
| Effector T cells | CD4^+^ CD45RA^+^ CCR7^-^ / CD8^+^ CD45RA^+^ CCR7^-^ |
| Central memory T cells | CD4^+^ CD45RO^+^ CCR7^+^ / CD8^+^ CD45RO^+^ CCR7^+^ |
| Effector memory T cells | CD4^+^ CD45RO^+^ CCR7^-^ / CD8^+^ CD45RO^+^ CCR7^-^ |

**Table S2:** Cohort characteristics of controls and PD patients with PBMC samples available.

|  | **Control (n=21)** | **Parkinson's Disease (n=13)** | **P-value** |
| --- | --- | --- | --- |
| Age (mean±SD) | 58.96 ± 12.36 | 61.46 ± 5.38 | 0.81 |
| Sex (female, %) | 11 (52.38) | 7 (53.85) | >0.99 |
| BMI (mean±SD) | 26.55 ± 4.31 | 26.90 ± 5.55 | 0.85 |
| Difficulty swallowing (%) | 1 (5%) | 5 (39%) | **0.02** |
| Vomiting or nausea (%) # | 1 (5%) | 4 (33%) | **0.05** |
| Constipation in last month (%) | 3 (17.65) | 7 (53.85) | **0.02** |
| Incomplete bowel emptying (%) # | 0 | 1 (8%) | 0.39 |
| **Bowel Pattern in the last 12 months#** | | | |
| Normal (%) | 20 (95%) | 5 (42%) | **0.0006** |
| Alternating (%) | 0 | 1 (8%) | 0.36 |
| Constipated (%) | 1 (5%) | 6 (50%) | 0.005 |
| **Smoking history#** | | | |
| Non-smoker | 14 (67%) | 5 (42%) | 0.27 |
| Ex-smoker | 6 (29%) | 7 (58%) | 0.14 |
| Current smoker | 1 (5%) | 0 | >0.99 |

*#data not reported for all; body mass index (BMI); standard deviation (SD); Constipation defined as answering yes to the question “less than 3 bowel movements a week or having to strain to pass a stool”.*

**Table S3.** Clinical characteristics and medication of Parkinson’s disease (PD) participants at the time of sample collection. Gastrointestinal (GI). Active ingredients for medications managing PD symptoms included levodopa, carbidopa, entacapone, pramipexole, and benserazide. Active ingredients for the management of GI symptoms included docusate sodium and sennosides, domperidone, and rabeprazole sodium.

**Table S4.** Spearman correlation results between gut microbiome species, PBMC immune markers and gastrointestinal (GI) symptoms in PD (Case) and Control groups. The table reports the correlation coefficient (rho), and adjusted p‑value (Benjamini–Hochberg FDR), with significance indicated by asterisks (* p < 0.05; ** p < 0.01; *** p < 0.001). Species names were extracted at the species level from the full taxonomic lineage. Positive rho values indicate positive associations; negative rho values indicate inverse associations.

| **Group** | **Species** | **Immune_marker** | **Rho** | **Adjusted P-vaue** | **Significance** |
| --- | --- | --- | --- | --- | --- |
| Control | Turicibacter_sanguinis | CD4. | 0.076 | 0.773 |  |
| Control | Turicibacter_sanguinis | CD4._Central_Memory | 0.179 | 0.491 |  |
| Control | Turicibacter_sanguinis | CD4._Effector | -0.211 | 0.415 |  |
| Control | Turicibacter_sanguinis | CD4._Effector_Memory | 0.255 | 0.322 |  |
| Control | Turicibacter_sanguinis | CD4._Gut_Homing | 0.025 | 0.928 |  |
| Control | Turicibacter_sanguinis | CD4._Naive | 0.076 | 0.773 |  |
| Control | Turicibacter_sanguinis | CD8. | 0.145 | 0.579 |  |
| Control | Turicibacter_sanguinis | CD8._Central_Memory | 0.093 | 0.723 |  |
| Control | Turicibacter_sanguinis | CD8._Effector | 0.047 | 0.861 |  |
| Control | Turicibacter_sanguinis | CD8._Effector_Memory | 0.277 | 0.281 |  |
| Control | Turicibacter_sanguinis | CD8._Gut_Homing | -0.108 | 0.680 |  |
| Control | Turicibacter_sanguinis | CD8._Naive | 0.032 | 0.906 |  |
| PD | Turicibacter_sanguinis | CD4. | 0.429 | 0.146 |  |
| PD | Turicibacter_sanguinis | CD4._Central_Memory | 0.374 | 0.209 |  |
| PD | Turicibacter_sanguinis | CD4._Effector | 0.604 | 0.032 | * |
| PD | Turicibacter_sanguinis | CD4._Effector_Memory | 0.385 | 0.196 |  |
| PD | Turicibacter_sanguinis | CD4._Gut_Homing | 0.698 | 0.010 | * |
| PD | Turicibacter_sanguinis | CD4._Naive | 0.368 | 0.217 |  |
| PD | Turicibacter_sanguinis | CD8. | 0.841 | 0.001 | *** |
| PD | Turicibacter_sanguinis | CD8._Central_Memory | 0.549 | 0.055 |  |
| PD | Turicibacter_sanguinis | CD8._Effector | 0.764 | 0.004 | ** |
| PD | Turicibacter_sanguinis | CD8._Effector_Memory | 0.632 | 0.024 | * |
| PD | Turicibacter_sanguinis | CD8._Gut_Homing | 0.764 | 0.004 | ** |
| PD | Turicibacter_sanguinis | CD8._Naive | 0.632 | 0.024 | * |
| Control | Sutterella_sp_AM11_39 | CD4. | -0.216 | 0.404 |  |
| Control | Sutterella_sp_AM11_39 | CD4._Central_Memory | -0.152 | 0.559 |  |
| Control | Sutterella_sp_AM11_39 | CD4._Effector | -0.507 | 0.040 | * |
| Control | Sutterella_sp_AM11_39 | CD4._Effector_Memory | 0.032 | 0.906 |  |
| Control | Sutterella_sp_AM11_39 | CD4._Gut_Homing | -0.174 | 0.503 |  |
| Control | Sutterella_sp_AM11_39 | CD4._Naive | -0.145 | 0.579 |  |
| Control | Sutterella_sp_AM11_39 | CD8. | 0.110 | 0.673 |  |
| Control | Sutterella_sp_AM11_39 | CD8._Central_Memory | -0.044 | 0.869 |  |
| Control | Sutterella_sp_AM11_39 | CD8._Effector | -0.027 | 0.921 |  |
| Control | Sutterella_sp_AM11_39 | CD8._Effector_Memory | 0.152 | 0.559 |  |
| Control | Sutterella_sp_AM11_39 | CD8._Gut_Homing | -0.179 | 0.491 |  |
| Control | Sutterella_sp_AM11_39 | CD8._Naive | -0.022 | 0.936 |  |
| PD | Sutterella_sp_AM11_39 | CD4. | 0.637 | 0.022 | * |
| PD | Sutterella_sp_AM11_39 | CD4._Central_Memory | 0.549 | 0.055 |  |
| PD | Sutterella_sp_AM11_39 | CD4._Effector | 0.604 | 0.032 | * |
| PD | Sutterella_sp_AM11_39 | CD4._Effector_Memory | 0.341 | 0.255 |  |
| PD | Sutterella_sp_AM11_39 | CD4._Gut_Homing | 0.852 | 0.000 | *** |
| PD | Sutterella_sp_AM11_39 | CD4._Naive | 0.588 | 0.038 | * |
| PD | Sutterella_sp_AM11_39 | CD8. | 0.665 | 0.016 | * |
| PD | Sutterella_sp_AM11_39 | CD8._Central_Memory | 0.648 | 0.020 | * |
| PD | Sutterella_sp_AM11_39 | CD8._Effector | 0.648 | 0.020 | * |
| PD | Sutterella_sp_AM11_39 | CD8._Effector_Memory | 0.335 | 0.263 |  |
| PD | Sutterella_sp_AM11_39 | CD8._Gut_Homing | 0.753 | 0.004 | ** |
| PD | Sutterella_sp_AM11_39 | CD8._Naive | 0.731 | 0.006 | ** |
| Control | GGB9788_SGB15411 | CD4. | 0.179 | 0.491 |  |
| Control | GGB9788_SGB15411 | CD4._Central_Memory | 0.316 | 0.216 |  |
| Control | GGB9788_SGB15411 | CD4._Effector | 0.010 | 0.974 |  |
| Control | GGB9788_SGB15411 | CD4._Effector_Memory | 0.314 | 0.220 |  |
| Control | GGB9788_SGB15411 | CD4._Gut_Homing | 0.096 | 0.715 |  |
| Control | GGB9788_SGB15411 | CD4._Naive | 0.157 | 0.547 |  |
| Control | GGB9788_SGB15411 | CD8. | 0.145 | 0.579 |  |
| Control | GGB9788_SGB15411 | CD8._Central_Memory | 0.172 | 0.509 |  |
| Control | GGB9788_SGB15411 | CD8._Effector | 0.309 | 0.227 |  |
| Control | GGB9788_SGB15411 | CD8._Effector_Memory | 0.289 | 0.259 |  |
| Control | GGB9788_SGB15411 | CD8._Gut_Homing | -0.096 | 0.715 |  |
| Control | GGB9788_SGB15411 | CD8._Naive | 0.071 | 0.787 |  |
| PD | GGB9788_SGB15411 | CD4. | 0.835 | 0.001 | *** |
| PD | GGB9788_SGB15411 | CD4._Central_Memory | 0.692 | 0.011 | * |
| PD | GGB9788_SGB15411 | CD4._Effector | 0.473 | 0.106 |  |
| PD | GGB9788_SGB15411 | CD4._Effector_Memory | 0.390 | 0.189 |  |
| PD | GGB9788_SGB15411 | CD4._Gut_Homing | 0.643 | 0.021 | * |
| PD | GGB9788_SGB15411 | CD4._Naive | 0.775 | 0.003 | ** |
| PD | GGB9788_SGB15411 | CD8. | 0.604 | 0.032 | * |
| PD | GGB9788_SGB15411 | CD8._Central_Memory | 0.758 | 0.004 | ** |
| PD | GGB9788_SGB15411 | CD8._Effector | 0.533 | 0.064 |  |
| PD | GGB9788_SGB15411 | CD8._Effector_Memory | 0.390 | 0.189 |  |
| PD | GGB9788_SGB15411 | CD8._Gut_Homing | 0.621 | 0.027 | * |
| PD | GGB9788_SGB15411 | CD8._Naive | 0.813 | 0.001 | ** |
| Control | GGB9364_SGB14339 | CD4. | -0.027 | 0.921 |  |
| Control | GGB9364_SGB14339 | CD4._Central_Memory | 0.047 | 0.861 |  |
| Control | GGB9364_SGB14339 | CD4._Effector | -0.348 | 0.171 |  |
| Control | GGB9364_SGB14339 | CD4._Effector_Memory | 0.191 | 0.461 |  |
| Control | GGB9364_SGB14339 | CD4._Gut_Homing | -0.081 | 0.758 |  |
| Control | GGB9364_SGB14339 | CD4._Naive | -0.020 | 0.943 |  |
| Control | GGB9364_SGB14339 | CD8. | 0.179 | 0.491 |  |
| Control | GGB9364_SGB14339 | CD8._Central_Memory | 0.127 | 0.625 |  |
| Control | GGB9364_SGB14339 | CD8._Effector | 0.005 | 0.989 |  |
| Control | GGB9364_SGB14339 | CD8._Effector_Memory | 0.311 | 0.223 |  |
| Control | GGB9364_SGB14339 | CD8._Gut_Homing | -0.135 | 0.605 |  |
| Control | GGB9364_SGB14339 | CD8._Naive | 0.017 | 0.951 |  |
| PD | GGB9364_SGB14339 | CD4. | 0.401 | 0.176 |  |
| PD | GGB9364_SGB14339 | CD4._Central_Memory | 0.352 | 0.239 |  |
| PD | GGB9364_SGB14339 | CD4._Effector | 0.582 | 0.040 | * |
| PD | GGB9364_SGB14339 | CD4._Effector_Memory | 0.368 | 0.217 |  |
| PD | GGB9364_SGB14339 | CD4._Gut_Homing | 0.659 | 0.017 | * |
| PD | GGB9364_SGB14339 | CD4._Naive | 0.341 | 0.255 |  |
| PD | GGB9364_SGB14339 | CD8. | 0.846 | 0.000 | *** |
| PD | GGB9364_SGB14339 | CD8._Central_Memory | 0.533 | 0.064 |  |
| PD | GGB9364_SGB14339 | CD8._Effector | 0.758 | 0.004 | ** |
| PD | GGB9364_SGB14339 | CD8._Effector_Memory | 0.626 | 0.025 | * |
| PD | GGB9364_SGB14339 | CD8._Gut_Homing | 0.747 | 0.005 | ** |
| PD | GGB9364_SGB14339 | CD8._Naive | 0.610 | 0.030 | * |
| Control | GGB33512_SGB15201 | CD4. | -0.147 | 0.572 |  |
| Control | GGB33512_SGB15201 | CD4._Central_Memory | -0.100 | 0.701 |  |
| Control | GGB33512_SGB15201 | CD4._Effector | 0.017 | 0.951 |  |
| Control | GGB33512_SGB15201 | CD4._Effector_Memory | -0.022 | 0.936 |  |
| Control | GGB33512_SGB15201 | CD4._Gut_Homing | -0.093 | 0.723 |  |
| Control | GGB33512_SGB15201 | CD4._Naive | -0.211 | 0.415 |  |
| Control | GGB33512_SGB15201 | CD8. | -0.029 | 0.913 |  |
| Control | GGB33512_SGB15201 | CD8._Central_Memory | -0.110 | 0.673 |  |
| Control | GGB33512_SGB15201 | CD8._Effector | 0.304 | 0.235 |  |
| Control | GGB33512_SGB15201 | CD8._Effector_Memory | 0.007 | 0.981 |  |
| Control | GGB33512_SGB15201 | CD8._Gut_Homing | -0.032 | 0.906 |  |
| Control | GGB33512_SGB15201 | CD8._Naive | -0.176 | 0.497 |  |
| PD | GGB33512_SGB15201 | CD4. | 0.582 | 0.040 | * |
| PD | GGB33512_SGB15201 | CD4._Central_Memory | 0.516 | 0.074 |  |
| PD | GGB33512_SGB15201 | CD4._Effector | 0.813 | 0.001 | ** |
| PD | GGB33512_SGB15201 | CD4._Effector_Memory | 0.516 | 0.074 |  |
| PD | GGB33512_SGB15201 | CD4._Gut_Homing | 0.599 | 0.034 | * |
| PD | GGB33512_SGB15201 | CD4._Naive | 0.401 | 0.176 |  |
| PD | GGB33512_SGB15201 | CD8. | 0.830 | 0.001 | *** |
| PD | GGB33512_SGB15201 | CD8._Central_Memory | 0.604 | 0.032 | * |
| PD | GGB33512_SGB15201 | CD8._Effector | 0.896 | 0.000 | *** |
| PD | GGB33512_SGB15201 | CD8._Effector_Memory | 0.544 | 0.058 |  |
| PD | GGB33512_SGB15201 | CD8._Gut_Homing | 0.643 | 0.021 | * |
| PD | GGB33512_SGB15201 | CD8._Naive | 0.621 | 0.027 | * |
| Control | Enterocloster_bolteae | CD4. | -0.353 | 0.165 |  |
| Control | Enterocloster_bolteae | CD4._Central_Memory | -0.365 | 0.150 |  |
| Control | Enterocloster_bolteae | CD4._Effector | -0.309 | 0.227 |  |
| Control | Enterocloster_bolteae | CD4._Effector_Memory | -0.314 | 0.220 |  |
| Control | Enterocloster_bolteae | CD4._Gut_Homing | -0.294 | 0.251 |  |
| Control | Enterocloster_bolteae | CD4._Naive | -0.390 | 0.123 |  |
| Control | Enterocloster_bolteae | CD8. | -0.463 | 0.063 |  |
| Control | Enterocloster_bolteae | CD8._Central_Memory | -0.319 | 0.212 |  |
| Control | Enterocloster_bolteae | CD8._Effector | -0.811 | 0.000 | *** |
| Control | Enterocloster_bolteae | CD8._Effector_Memory | -0.400 | 0.113 |  |
| Control | Enterocloster_bolteae | CD8._Gut_Homing | -0.267 | 0.299 |  |
| Control | Enterocloster_bolteae | CD8._Naive | -0.375 | 0.139 |  |
| PD | Enterocloster_bolteae | CD4. | 0.055 | 0.863 |  |
| PD | Enterocloster_bolteae | CD4._Central_Memory | -0.011 | 0.978 |  |
| PD | Enterocloster_bolteae | CD4._Effector | 0.451 | 0.125 |  |
| PD | Enterocloster_bolteae | CD4._Effector_Memory | 0.291 | 0.334 |  |
| PD | Enterocloster_bolteae | CD4._Gut_Homing | 0.280 | 0.353 |  |
| PD | Enterocloster_bolteae | CD4._Naive | 0.060 | 0.849 |  |
| PD | Enterocloster_bolteae | CD8. | 0.198 | 0.517 |  |
| PD | Enterocloster_bolteae | CD8._Central_Memory | 0.132 | 0.669 |  |
| PD | Enterocloster_bolteae | CD8._Effector | 0.313 | 0.297 |  |
| PD | Enterocloster_bolteae | CD8._Effector_Memory | 0.247 | 0.415 |  |
| PD | Enterocloster_bolteae | CD8._Gut_Homing | 0.159 | 0.604 |  |
| PD | Enterocloster_bolteae | CD8._Naive | 0.077 | 0.807 |  |
| Control | Dialister_hominis | CD4. | -0.027 | 0.921 |  |
| Control | Dialister_hominis | CD4._Central_Memory | 0.047 | 0.861 |  |
| Control | Dialister_hominis | CD4._Effector | -0.348 | 0.171 |  |
| Control | Dialister_hominis | CD4._Effector_Memory | 0.191 | 0.461 |  |
| Control | Dialister_hominis | CD4._Gut_Homing | -0.081 | 0.758 |  |
| Control | Dialister_hominis | CD4._Naive | -0.020 | 0.943 |  |
| Control | Dialister_hominis | CD8. | 0.179 | 0.491 |  |
| Control | Dialister_hominis | CD8._Central_Memory | 0.127 | 0.625 |  |
| Control | Dialister_hominis | CD8._Effector | 0.005 | 0.989 |  |
| Control | Dialister_hominis | CD8._Effector_Memory | 0.311 | 0.223 |  |
| Control | Dialister_hominis | CD8._Gut_Homing | -0.135 | 0.605 |  |
| Control | Dialister_hominis | CD8._Naive | 0.017 | 0.951 |  |
| PD | Dialister_hominis | CD4. | 0.582 | 0.040 | * |
| PD | Dialister_hominis | CD4._Central_Memory | 0.610 | 0.030 | * |
| PD | Dialister_hominis | CD4._Effector | 0.187 | 0.541 |  |
| PD | Dialister_hominis | CD4._Effector_Memory | 0.011 | 0.978 |  |
| PD | Dialister_hominis | CD4._Gut_Homing | 0.813 | 0.001 | ** |
| PD | Dialister_hominis | CD4._Naive | 0.764 | 0.004 | ** |
| PD | Dialister_hominis | CD8. | 0.374 | 0.209 |  |
| PD | Dialister_hominis | CD8._Central_Memory | 0.725 | 0.007 | ** |
| PD | Dialister_hominis | CD8._Effector | 0.308 | 0.306 |  |
| PD | Dialister_hominis | CD8._Effector_Memory | 0.187 | 0.541 |  |
| PD | Dialister_hominis | CD8._Gut_Homing | 0.742 | 0.005 | ** |
| PD | Dialister_hominis | CD8._Naive | 0.830 | 0.001 | *** |
| Control | Akkermansia_sp_KLE1798 | CD4. | -0.027 | 0.921 |  |
| Control | Akkermansia_sp_KLE1798 | CD4._Central_Memory | 0.047 | 0.861 |  |
| Control | Akkermansia_sp_KLE1798 | CD4._Effector | -0.348 | 0.171 |  |
| Control | Akkermansia_sp_KLE1798 | CD4._Effector_Memory | 0.191 | 0.461 |  |
| Control | Akkermansia_sp_KLE1798 | CD4._Gut_Homing | -0.081 | 0.758 |  |
| Control | Akkermansia_sp_KLE1798 | CD4._Naive | -0.020 | 0.943 |  |
| Control | Akkermansia_sp_KLE1798 | CD8. | 0.179 | 0.491 |  |
| Control | Akkermansia_sp_KLE1798 | CD8._Central_Memory | 0.127 | 0.625 |  |
| Control | Akkermansia_sp_KLE1798 | CD8._Effector | 0.005 | 0.989 |  |
| Control | Akkermansia_sp_KLE1798 | CD8._Effector_Memory | 0.311 | 0.223 |  |
| Control | Akkermansia_sp_KLE1798 | CD8._Gut_Homing | -0.135 | 0.605 |  |
| Control | Akkermansia_sp_KLE1798 | CD8._Naive | 0.017 | 0.951 |  |
| PD | Akkermansia_sp_KLE1798 | CD4. | 0.555 | 0.053 |  |
| PD | Akkermansia_sp_KLE1798 | CD4._Central_Memory | 0.456 | 0.120 |  |
| PD | Akkermansia_sp_KLE1798 | CD4._Effector | 0.863 | 0.000 | *** |
| PD | Akkermansia_sp_KLE1798 | CD4._Effector_Memory | 0.753 | 0.004 | ** |
| PD | Akkermansia_sp_KLE1798 | CD4._Gut_Homing | 0.467 | 0.110 |  |
| PD | Akkermansia_sp_KLE1798 | CD4._Naive | 0.280 | 0.353 |  |
| PD | Akkermansia_sp_KLE1798 | CD8. | 0.577 | 0.043 | * |
| PD | Akkermansia_sp_KLE1798 | CD8._Central_Memory | 0.429 | 0.146 |  |
| PD | Akkermansia_sp_KLE1798 | CD8._Effector | 0.720 | 0.007 | ** |
| PD | Akkermansia_sp_KLE1798 | CD8._Effector_Memory | 0.489 | 0.093 |  |
| PD | Akkermansia_sp_KLE1798 | CD8._Gut_Homing | 0.429 | 0.146 |  |
| PD | Akkermansia_sp_KLE1798 | CD8._Naive | 0.357 | 0.232 |  |
| PD | Veillonella_atypica | Vomiting_or_nausea | 0.759 | 0.812 | ** |
| PD | Ruminococcus_gnavus | Difficulty_swallowing | 0.717 | 0.815 | ** |
| PD | Pseudoflavonifractor_gallinarum | Bowel_pattern | 0.814 | 0.895 | ** |
| PD | Phocaeicola_coprophilus | Incomplete_bowel_emptying | -0.710 | 0.375 | ** |
| PD | Odoribacter_laneus | Incomplete_bowel_emptying | -0.710 | 0.375 | ** |
| PD | Muribaculum_gordoncarteri | Incomplete_bowel_emptying | -0.710 | 0.375 | ** |
| PD | Intestinimonas_massiliensis | Incomplete_bowel_emptying | 0.661 | 0.375 | * |
| PD | Intestinimonas_massiliensis | Vomiting_or_nausea | -0.710 | 0.812 | ** |
| PD | GGB9818_SGB15459 | Constipation | -0.724 | 0.573 | ** |
| PD | GGB9788_SGB15411 | Bowel_pattern | 0.724 | 0.895 | * |
| PD | GGB9788_SGB15411 | Constipation | 0.772 | 0.573 | ** |
| PD | GGB9637_SGB15111 | Bowel_pattern | 0.633 | 0.895 | * |
| PD | GGB9637_SGB15111 | Constipation | 0.724 | 0.573 | ** |
| PD | GGB9627_SGB15081 | Vomiting_or_nausea | -0.759 | 0.812 | ** |
| PD | GGB9545_SGB14952 | Incomplete_bowel_emptying | -0.710 | 0.375 | ** |
| PD | GGB4285_SGB5849 | Incomplete_bowel_emptying | -0.710 | 0.375 | ** |
| PD | GGB3548_SGB4744 | Incomplete_bowel_emptying | -0.710 | 0.375 | ** |
| PD | GGB2652_SGB3573 | Incomplete_bowel_emptying | -0.808 | 0.375 | ** |
| PD | GGB2652_SGB3573 | Vomiting_or_nausea | 0.612 | 0.812 | * |
| PD | GGB1543_SGB2126 | Incomplete_bowel_emptying | -0.710 | 0.375 | ** |
| PD | Enterocloster_asparagiformis | Incomplete_bowel_emptying | -0.710 | 0.375 | ** |
| PD | Desulfovibrio_SGB101145 | Incomplete_bowel_emptying | -0.710 | 0.375 | ** |
| PD | Collinsella_stercoris | Incomplete_bowel_emptying | -0.710 | 0.375 | ** |
| PD | Bacteroides_cutis | Incomplete_bowel_emptying | -0.710 | 0.375 | ** |
| PD | Allisonella_histaminiformans | Incomplete_bowel_emptying | -0.857 | 0.245 | *** |
| PD | Allisonella_histaminiformans | Vomiting_or_nausea | 0.661 | 0.812 | * |
| PD | Alistipes_sp_An31A | Incomplete_bowel_emptying | -0.857 | 0.245 | *** |
| PD | Alistipes_sp_An31A | Vomiting_or_nausea | 0.661 | 0.812 | * |
| PD | Alistipes_finegoldii | Incomplete_bowel_emptying | 0.710 | 0.375 | ** |
| PD | Alistipes_finegoldii | Vomiting_or_nausea | -0.710 | 0.812 | ** |


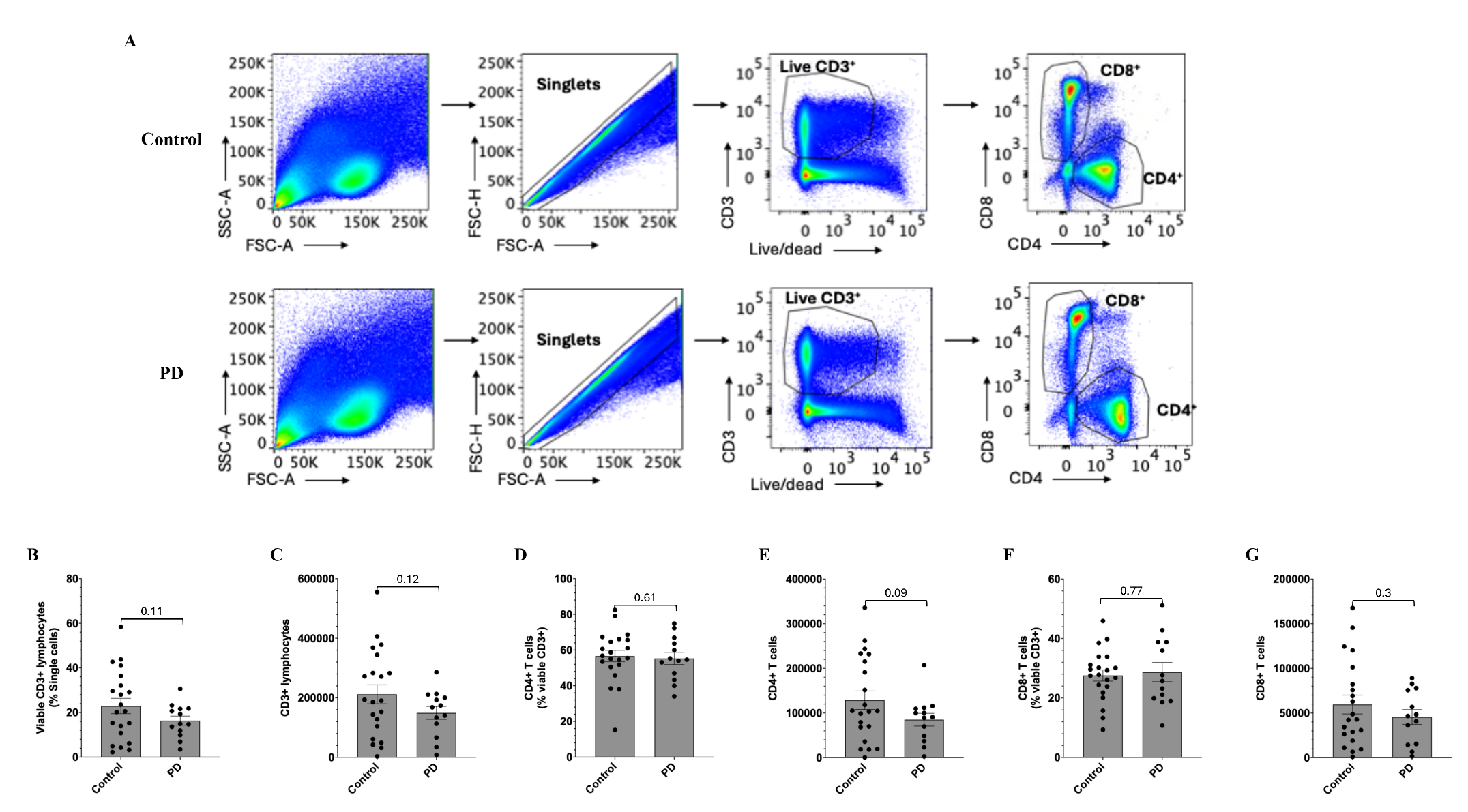


**Figure S1.** Lymphocyte populations in controls and Parkinson’s Disease (PD) patients. Isolated PBMCs from controls and PD patients **A)** were analysed by flow cytometry using surface marker expression. Viable CD3+ lymphocytes (fixable viability stain negative) were assessed as both **B)** a proportion of single cells and **C)** total cell number. CD4^+^ T helper cells were analysed as **D)** a proportion of viable CD3^+^ and **E)** total cell number, as were CD8^+^ cytotoxic T cells as a **F)** proportion of viable CD3^+^ and **G)** total cell number. n=21 controls, n=13 PD, data presented as mean±SEM.


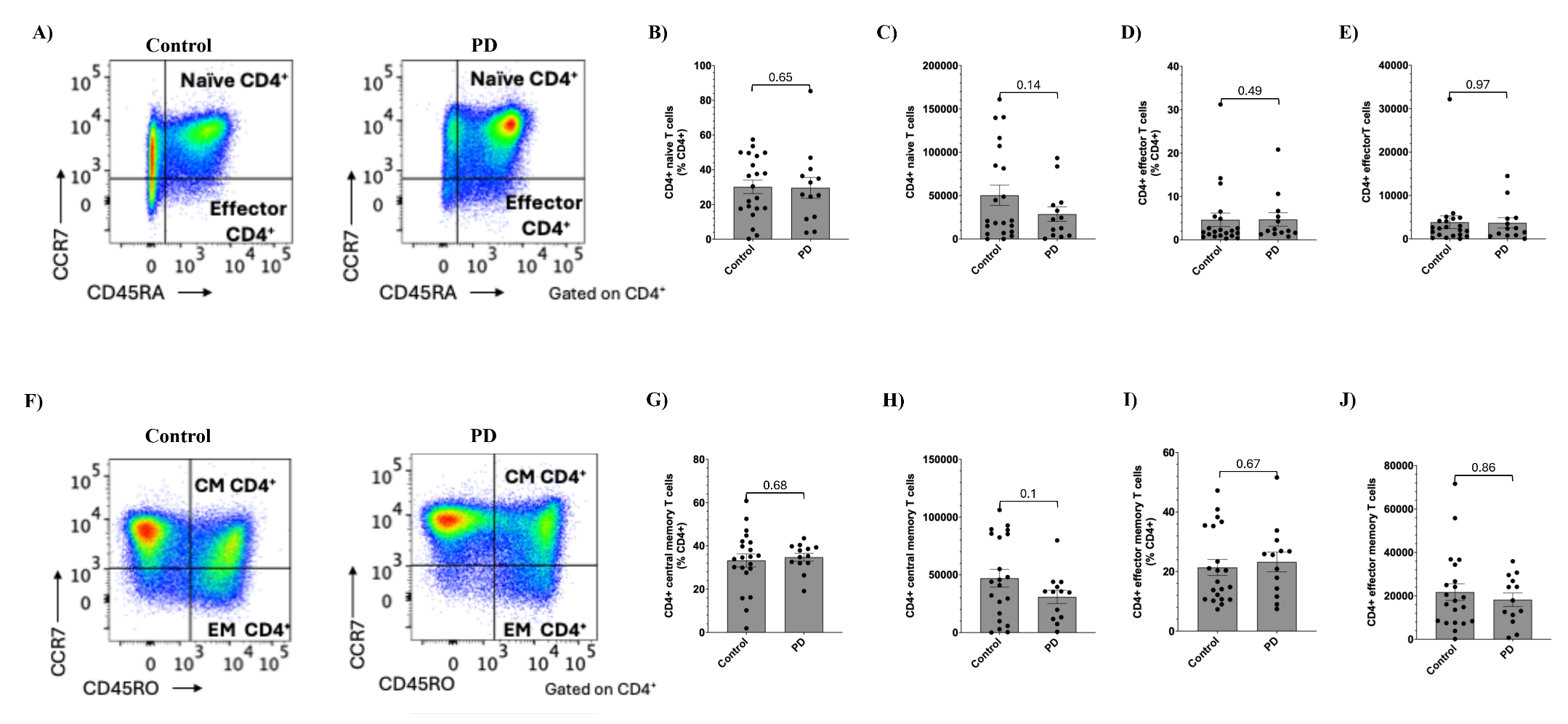


**Figure S2.** CD4^+^ naïve, effector and memory populations in peripheral blood from controls and Parkinson’s Disease (PD) patients. **A)** Naïve and effector CD4^+^ lymphocytes were gated based on the expression of CD45RA and CCR7. CD4^+^ naïve (CD45RA^+^ CCR7^+^) lymphocytes were assessed as both **B)** a proportion of the total CD4^+^ population and **C)** total cell number, while CD4^+^ effector lymphocytes (CD45RA^+^ CCR7^-^) were also assessed as both **D)** a proportion of the total CD4^+^ population and **E)** total cell number. **F)** Central memory and effector memory CD4^+^ lymphocytes were gated based on the expression of CD45RO and CCR7. CD4^+^ central memory (CM, CD45RO^+^ CCR7^+^) lymphocytes were assessed as both **G)** a proportion of the total CD4^+^ population and **H)** total cell number, while CD4^+^ effector memory (EM, CD45RO^+^ CCR7^-^) lymphocytes were also assessed as both **I)** a proportion of the total CD4^+^ population and **J)** total cell number. n=21 controls, n=13 PD, data presented as mean±SEM.


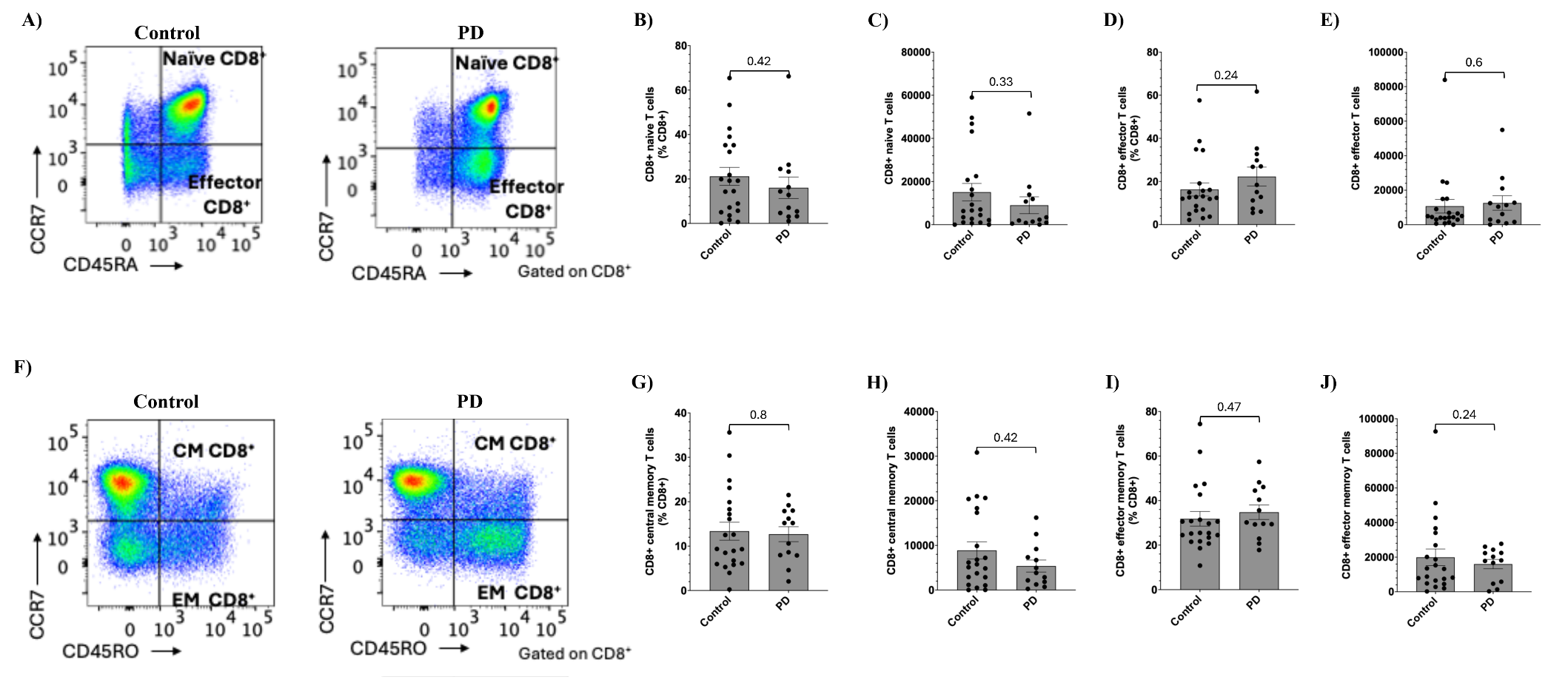


**Figure S3.** CD8^+^ naïve, effector and memory populations in peripheral blood from controls and Parkinson’s Disease (PD) patients. **A)** Naïve and effector CD8^+^ lymphocytes were gated based on the expression of CD45RA and CCR7. CD8^+^ naïve (CD45RA^+^ CCR7^+^) lymphocytes were assessed as both **B)** a proportion of the total CD8^+^ population and **C)** total cell number, while CD8^+^ effector lymphocytes (CD45RA^+^ CCR7^-^) were also assessed as both **D)** a proportion of the total CD8^+^ population and **E)** total cell number. **F)** Central memory and effector memory effector CD8^+^ lymphocytes were gated based on the expression of CD45RO and CCR7. CD8^+^ central memory (CM, CD45RO^+^ CCR7^+^) lymphocytes were assessed as both **G)** a proportion of the total CD8^+^ population and **H)** total cell number, while CD8^+^ effector memory (EM, CD45RO^+^ CCR7^-^) lymphocytes were also assessed as both **I)** a proportion of the total CD8^+^ population and **J)** total cell number. n=21 controls, n=13 PD, data presented as mean±SEM.
